# Cytokine Storm Syndrome Triggered by Extracorporeal Membrane Oxygenation in Pediatric Patients

**DOI:** 10.3390/children10061052

**Published:** 2023-06-13

**Authors:** Daniel D. Reiff, Randy Q. Cron

**Affiliations:** Department of Pediatrics, Division of Rheumatology, University of Alabama at Birmingham, Birmingham, AL 35233, USA; danielreiff@uabmc.edu

**Keywords:** cytokine storm, inflammation, cardiopulmonary bypass, extracorporeal membrane oxygenation, cytokine

## Abstract

Cytokine storm syndrome (CSS) is a serious and potentially life-threatening condition caused by severe systemic inflammation, immune activation, and a positive feedback loop of cytokine release. Typically triggered by systemic infection, malignancy, monogenic or rheumatic disease, similar patterns of hyper-inflammation have been seen in patients undergoing cardiopulmonary bypass (CPB) and in patients treated with extracorporeal membrane oxygenation (ECMO). Typical treatments used for the prevention and treatment of CPB/ECMO-induced hyper-inflammation have not been shown to be substantially effective. Two patients suffering from ECMO-related CSS were identified by their severe hyper-inflammatory profile and life-threatening sequelae of vasodilatory shock and respiratory failure. Anakinra, an interleukin-1 receptor antagonist, was employed as specific cytokine-directed therapy for the treatment of CSS in these two patients to good effect, with significant improvement in hyper-inflammation and cardiorespiratory status. The use of cytokine-directed therapies in CPB/ECMO-related CSS has great potential to improve the treatment and outcomes of this serious condition.

## 1. Introduction

Cytokine storm syndrome (CSS) is a serious and potentially life-threatening condition caused by severe systemic inflammation, immune system activation, and a positive feedback loop of cytokine release. This pattern of hyper-inflammation can result from many different triggers, including but not limited to monogenic diseases, systemic viral infections, chimeric antigen receptor (CAR) T-cell therapy, in response to rheumatic disease, and with genetic immunodeficiencies [1,2]. The resultant release of pro-inflammatory cytokines interleukin-1 (IL-1), IL-6, IL-18, interferon-γ, and tumor necrosis factor (TNF) leads to fever, rash, neuropsychiatric symptoms, hypotensive shock, end-organ damage, and even death in severely affected patients [1,2].

Cardiopulmonary bypass (CPB) and extracorporeal membrane oxygenation (ECMO) have been increasingly used and to good effect in the treatment and support of cardio-respiratory failure, but an inflammatory response similar to CSS has been reported to result from these treatments [3]. Comparable elevations in pro-inflammatory cytokines and activation of the complement system and coagulation cascade can likewise lead to end-organ damage and increased morbidity and mortality in the setting of CPB and ECMO [3]. Current treatments for CPB/ECMO-induced hyper-inflammation with steroid therapy or hemadsorption filters are limited in their use and efficacy. Herein, we report on two pediatric cases of ECMO-induced CSS and the use of cytokine-directed therapies as treatment. We also discuss the current understanding of CSS in CPB and ECMO, current management options, and the potential utility of targeted anti-cytokine therapies in the treatment of this severe condition.

## 2. Materials and Methods

The patients’ electronic medical records were reviewed, and laboratory values, clinical course, therapeutics, outcomes, and post-hospitalization care were abstracted and analyzed. All information was de-identified following an Institutional Review Board-approved protocol. This study using de-identified patient data was conducted using the University of Alabama at Birmingham Institutional Review Board-approved protocol, number 300007457. The tables and calculations were created and performed using Microsoft Excel, respectively.

## 3. Case One

A 38-week gestational age female was born via C-section due to failure to progress in the setting of known hypoplastic left heart syndrome. The pregnancy was complicated by intrauterine growth restriction (IUGR) with concern for Turner syndrome, confirmed via microarray shortly after birth. The patient was stabilized on prostaglandin infusion and did not initially require any respiratory support. Due to her known genetic abnormality and IUGR, she was determined to be a poor candidate for surgical repair. During cardiac catheterization on day of life (DOL) 2, she was intubated and remained on mechanical ventilation while awaiting her surgical procedure. On DOL 7, she underwent an atrial septectomy with application of bilateral pulmonary artery bands, while awaiting transplantation. The patient remained relatively stable while awaiting transplant, despite continued systolic heart failure and respiratory failure, and was slowly weaned off positive pressure ventilation by DOL 32. However, on DOL 41, she acutely decompensated with worsening respiratory failure, and with concern for impending cardiac arrest, the patient was placed on venous-arterial (VA) ECMO. Prior to the ECMO initiation, the patient did not have any infectious signs or symptoms and was afebrile.

Shortly after the initiation of ECMO, routine laboratory findings showed significant abnormalities in inflammatory and CSS markers (Table 1). Platelet count, white blood cell count, and hemoglobin dropped quickly upon ECMO initiation with a large increase in ferritin and liver enzyme levels. Fibrinogen levels quickly fell as well.

To aid in the diagnosis of CSS, two scoring systems were calculated—the HScore and HLH-2004 criteria—at the time of anakinra initiation (Table 2) [4,5]. This patient met three of the HLH-2004 criteria with cytopenia of two cell lines, low fibrinogen, and hyperferritinemia. The fever was difficult to assess, as ECMO circuits exposed blood to ambient temperature and can make the absence of fever an unreliable measure of the clinical status. Similarly, natural killer (NK) cell function and sCD25/soluble interleukin-2 receptor α were not measured in this patient. The patient’s cytopenias, ferritin level, fibrinogen level, and aspartate aminotransferase (AST) level all added to the HScore calculation in this case. An HScore of 123 corresponds to a 5–9% probability of hemophagocytic syndrome with 169 seen as the best cutoff score (sensitivity 93%, specificity 86%), but again, given the unreliability of fever and lack of bone marrow evaluation, the value of 123 was still seen as concerning for CSS in this specific clinical context [4,5].

Blood, tracheal aspirate, and peritoneal fluid cultures obtained with ECMO initiation remained negative, and the infant completed a 48-h antibiotic rule-out. No cytokine levels were obtained during this acute period. With enough concern for CSS and no obvious infectious causes, the patient was started on anakinra 50 mg twice daily (29 mg/kg/day) via intravenous injection on DOL 43. While on anakinra, lab abnormalities slowly improved with stabilization and eventual improvement of the cardiorespiratory status (Table 1). The patient was able to be decannulated from ECMO on DOL 46 and remained intubated on minimal ventilator settings.

Anakinra was continued after ECMO decannulation to treat residual inflammation. Ferritin decreased to 714.7 ng/mL at its nadir, liver enzymes improved to aspartate aminotransferase and alanine transaminase levels of 61 and 48 U/L, respectively, and fibrinogen levels normalized. However, despite the anakinra treatment and stabilization of lab values through the ECMO process, the patient continued to have intermittent code events, most notably on DOL 56, requiring intensive resuscitation, and on DOL 72, where she became acutely hypoxic and bradycardic with severe hypotension and gastrointestinal bleeding. On DOL 72, the decision was made to discontinue further resuscitation, and she passed from cardiac arrest shortly thereafter.

## 4. Case Two

A previously healthy 11-month-old Hispanic female presented to an outside hospital with a one-day history of shortness of breath, lack of appetite, and emesis. On arrival, she was noted to be in significant respiratory distress, requiring immediate intubation due to impeding respiratory collapse. A chest radiograph demonstrated cardiomegaly, and an echocardiogram showed significantly diminished cardiac function. With a pro-brain natriuretic peptide level of >70,000 pg/mL (normal < 178) and a troponin-T level of 65 ng/L (normal < 20), her clinical presentation was thought to be secondary to myocarditis-induced cardiogenic shock. After intubation, she required dopamine, milrinone, and epinephrine infusions, and was transferred to our tertiary care center. On arrival, she was cannulated onto VA-ECMO.

When a viral panel resulted positive for parainfluenza virus 3 and human rhinovirus/enterovirus, suspicion heightened for viral myocarditis. Initial bacterial cultures were negative, and the patient completed an antibiotic rule-out.

On ECMO initiation on hospital day (HD) 1, labs demonstrated signs of end-organ damage and inflammation, with liver enzyme elevation, acute kidney injury, and leukocytosis (Table 3).

Over the first three days of hospitalization and ECMO, her labs significantly worsened despite stabilization of her respiratory and cardiac status. With concern for worsening inflammation, a ferritin level was obtained and noted to be 8283.8 ng/mL. No cytokine testing was performed, but a soluble IL-2 receptor α level (marker of hyper-inflammation and hemophagocytic lymphohistiocytosis (HLH)) was noted to be 1362 U/mL (normal: 334–3026). This normal level was obtained on HD 7, so it is unclear if this represent treated/resolving CSS or a truly normal level that had been present since the ECMO onset. The HScore and HLH-2004 criteria were calculated at the time of anakinra initiation and are shown in Table 2. Four out of five HLH-2004 criteria were met with fever >38.5 °C, cytopenia of two cell lines, low fibrinogen, and hyperferritinemia. Her HScore reached a maximum of 156 with points for fever between 38.4 and 39.4 °C, fibrinogen level, cytopenias, ferritin level, and AST level. Rheumatology was consulted with concern for CSS, and anakinra 100 mg IV daily (14 mg/kg/day) was started on HD 4. After the initiation of anakinra, the patient’s lab values and clinical status improved to where she was decannulated from ECMO on HD 7 and extubated on HD 15.

Despite continued improvement in CSS labs post-ECMO decannulation, her cardiac function remained poor requiring a left ventricular assist device on HD 9. Anakinra was discontinued on HD 14. She was listed for an orthotopic heart transplant and underwent the procedure on hospital day 141. The procedure was well tolerated, and she was discharged on HD 158.

## 5. Discussion

Although ECMO and CPB have become increasingly beneficial in the management of respiratory and cardiac failure, these treatments have been associated with an increase in systemic inflammation via multiple pathways. As blood is exposed to the circuit and immune cells are exposed to foreign surfaces, the contact system is triggered, leading to multiple downstream effects influential in the signs and symptoms of CPB/ECMO-related hyper-inflammation [6]. Upon contact, the complement system is quickly triggered to activate the coagulation cascade, leading to the conversion of prothrombin to thrombin and subsequent clot formation [3]. Through complement activation, endothelial cells also increase their expression of selectins, leading to increased neutrophil activation and infiltration [7]. Endothelial cells and leukocytes are stimulated to release pro-inflammatory cytokines, leading to increased vascular permeability, endothelial injury, and a positive feedback loop of cytokine release [6,7]. Additionally, clamping of the aorta during these procedures is associated with ischemia-reperfusion injury, which has been shown to trigger a similar inflammatory response, driving neutrophil activation, reactive oxygen species, and pro-inflammatory cytokines [6,8].

Important pro-inflammatory cytokines implicated in this systemic response are interleukin-1 (IL-1), IL-6, IL-8, and tumor necrosis factor (TNF). In the setting of CPB/ECMO, higher levels of IL-6 and IL-8 have been associated with myocardial dysfunction and increased mortality risk, and similarly high levels of TNF and IL-6 have been seen in the neonatal and pediatric populations undergoing these treatments [9,10,11,12]. The anti-inflammatory cytokine IL-10 is also released and elevated in the setting of CPB/ECMO-related hyper-inflammation, but not to the level needed to balance the overall inflammatory response.

A similar hyper-inflammatory response can be seen in the context of systemic infection, after CAR T-cell therapy, in response to rheumatic disease, and with genetic immunodeficiencies. Originally thought of as distinct entities, these hyper-inflammatory responses are increasingly thought of under a single spectrum of disease: the cytokine storm syndrome umbrella [1]. Although the definition of CSS is still under debate and individual characteristics can differ in the setting of different triggers, CSS requires elevated levels of circulating cytokines, systemic inflammatory symptoms, secondary organ dysfunction, and improvement with anti-cytokine and anti-inflammatory treatments [2]. Symptoms include fever, headache, rash, myalgia, and neuropsychiatric changes, with findings of hypoxemia, cardiac or distributive shock, and respiratory or cardiac failure. End-organ damage results from tissue damage induced by cytokines, vascular occlusion, and perfusion-related injury. Scoring systems have been developed using various clinical and laboratory criteria in patients with CSS, two of the most utilized being the HScore and HLH-2004 criteria [4,5]. The HScore and HLH-2004 diagnostic/scoring criteria are described in Table 2, along with the score calculations from our patients’ data just after ECMO initiation. Accurate scores were unable to be obtained due to missing HLH-specific laboratory data and lack of bone marrow biopsy findings, so calculated scores may be underrepresenting the likelihood of CSS in these specific cases.

In many cases of patients requiring CPB/ECMO, it can be difficult to tease out the exact cause of hyper-inflammation and CSS. Infection, heart failure, prolonged cardiac arrest, and malignancies can all lead to hyper-inflammation in isolation, so when CPB/ECMO is utilized as treatment, it can be unclear which process is the main driver of the inflammatory process. In these two cases, we believe ECMO was the greatest contributor to CSS, as both patients had an acute increase in inflammation upon ECMO initiation, which persisted despite supportive therapies, only improving once anti-cytokine therapy was added. If the CSS was driven mainly by cardiac arrest and myocarditis, respectively, the inflammatory process would have been expected to improve upon supportive care and ECMO initiation as perfusion was restored.

In the pediatric rheumatology population, CSS is seen in the setting of monogenic disorders, systemic juvenile idiopathic arthritis, and systemic lupus erythematosus, and we were asked to help manage CSS response in the setting of severe infections as well. While the cytokine patterns can vary dependent on the trigger, similarly elevated levels of IL-1, IL-6, IL-10, and TNF are seen in these diseases, in addition to elevation of IL-18, IL-1 receptor antagonist, and interferon-γ [2]. With CSS and CPB/ECMO-related hyper-inflammation having similar symptoms, findings, and cytokine elevation patterns, it is likely that the hyper-inflammation seen in CPB/ECMO belongs under the same CSS umbrella, which may lead to new avenues of treatment.

Strategies currently employed to combat the hyper-inflammation seen in CPB/ECMO are limited, but options increase when viewed under the CSS umbrella. Steroid administration in the perioperative setting has been used to improve and/or prevent the hyper-inflammatory response to CPB/ECMO with mixed results. Although pretreatment with steroids has been shown to down-regulate pro-inflammatory cytokines and upregulate regulatory and anti-inflammatory cytokines in both adults and pediatric patients [13], no improvement in overall mortality has been shown with this intervention [13,14]. Another option currently used to control cytokines and hyper-inflammation is the use of hemadsorption filters attached to the CPB/ECMO circuit to filter and remove pro-inflammatory cytokines. Multiple randomized controlled trials have shown no difference between pre- and post-cardiopulmonary bypass levels of inflammatory cytokines within the first 24 h of treatment [15,16]. In the setting of ECMO, limited large-scale studies have been reported, with most data coming from small case studies or anecdotal observations. Studies have shown both a decrease and non-significant change in circulating cytokine levels, inflammatory markers, and mortality, leaving the effectiveness of cytokine adsorption during ECMO a continued question [6,7].

In pediatric rheumatology, the use of specific cytokine-directed therapeutics is routinely employed in the treatment of CSS, and we postulate that the use of these medications could be expanded to the treatment of CPB/ECMO-induced hyper-inflammation. In our two patients, anakinra was used to good effect with improvement in their inflammatory profile, vasodilatory shock, and respiratory failure. Anakinra is a recombinant IL-1 receptor antagonist, which has been shown to be safe and effective in the treatment of systemic juvenile idiopathic arthritis-related macrophage activation syndrome and CSS [17,18]. It has also been used in the management of pediatric infectious myocarditis, CSS related to viral infections, and COVID-19-related hyper-inflammation [17,18,19,20]. In our clinical practice, anakinra is used as first-line treatment for sHLH/MAS given its quick onset of action, subcutaneous administration option, short half-life (~6 h), and overall breadth of data in like use cases. Additionally, anakinra has been shown to have a wide dosing window with rare side effects [21,22,23]. Tocilizumab is an anti-IL-6 receptor monoclonal antibody that is used in CSS related to HLH and CAR T-cell therapy, and has been used extensively in CSS during the COVID-19 pandemic [24,25,26]. Less commonly used therapies include emapalumab (interferon-γ inhibitor) and Janus kinase inhibitors, reserved for specific cases of primary HLH and refractory cases of CSS. Studies employing these anti-cytokine-directed therapies should be pursued in the setting of CPB/ECMO-related hyper-inflammation.

## 6. Conclusions

Cytokine storm and its resultant hyper-inflammation can be serious and life-threatening sequelae of cardiopulmonary bypass and extracorporeal membrane oxygenation. The current use of steroids and cytokine filters for the prevention and treatment of cytokine storm has not been adequate to address this growing problem. As shown in these cases, specific cytokine-directed therapies like anakinra or tocilizumab are a new avenue for the treatment of CPB/ECMO-induced cytokine storm, and further studies should be employed to investigate these potential life-saving treatments.

## Figures and Tables

**Table 1:** Patient 1 Laboratory Results.

	DOL 40	DOL 41 ECMO Start	DOL 42	DOL 43 Anakinra Start	DOL 44	DOL 45	DOL 46 ECMO Stop	DOL 47
White Blood Cell count (7.05–14.68 × 10^3^ cells/uL)	6.91	4.13	4.32	4.99	4.85	7.32	6.71	16.45
Hemoglobin (9.2–11.4 g/dL)	14.5	9.9	9.5	10.9	11.9	11.5	10.5	14.8
Hematocrit (27.7–35.1%)	45.2	30.2	29.3	32.9	35.2	30.8	30.1	41.5
Platelet Count (140–440 × 10^3^ cells/uL)	236	65	106	63	50	55	38	29
Creatinine (0.1–0.36 mg/dL)	0.21	0.36	0.54	0.64	0.6	0.64	0.57	0.49
Aspartate Aminotransferase (20–67 U/L)	35.0	895.0	>4000.0	>4000.0	>4000.0	1332.0	357.0	90.0
Alanine Transaminase (5–33 U/L)	33.2	338.7	2158.5	2150.4	1698.4	1091.1	697.5	437.5
Ferritin (14–647.2 ng/mL)				>40,000.0	>40,000.0	67,211.8	18,560.0	4760.8
C Reactive Protein (0–0.5 mg/dL)		0.73			2.45	2.17	1.86	
Fibrinogen (156–400 mg/dL)		<60	115	154	143	142	153	153
Lactate Dehydrogenase (163–452 U/L)			24,017		14,004			

**Table 2:** Cytokine Storm Syndrome Scoring Systems.

	HLH-2004 [4]	HScore [5]
Patient 1	3/5	123
Patient 2	4/5	156
Diagnostic Criteria	5 out of 8 criteria:	Sum of all criteria scores:
	- Fever	Presence of immunosuppression, degree of fever, organomegaly, hemophagocytosis on bone marrow
	- Organomegaly	
	- Cytopenia of 2 or 3 lineages	Abnormalities in cell counts, ferritin, triglyceride, fibrinogen, or aspartase aminotransferase
	- High triglycerides or low fibrinogen	
	- Hemophagocytosis on bone marrow	
	- NK cell activity	Missing data reported as 0.
	- sCD25 level	If sum > 169, HScore is indicative of HLH.
	- Ferritin level	

NK—natural killer; HLH—hemophagocytic lymphohistiocytosis.

**Table 3:** Patient 2 Laboratory Results.

	Outside Hospital	HD 1 ECMO Start	HD 2	HD 3	HD 4 Anakinra Start	HD 5	HD 6	HD 7 ECMO Stop	HD 8	HD 14–16 Anakinra Stop
White Blood Cell count (6.48–13.02 ×10^3^ cells/uL)	15.84	21.87	14.19	10.59	8.49	10.51	12.79	14.94	10.89	9.17
Hemoglobin (10.5–13.5 g/dL)	8.8	8.4	10.3	9.7	9.1	8.7	10.7	13	11.3	9.9
Hematocrit (33–39%)	31.0	27.4	32	30.4	28.6	27	31.8	38.3	33.6	29.8
Platelet Count (140–440 × 10^3^ cells/uL)	370	296	68	67	77	64	43	88	88	266
Creatinine (0.1–0.36 mg/dL)	0.3	0.69	0.63	0.87	0.71	0.49	0.43	0.41	0.38	0.22
Aspartate Aminotransferase (20–67 U/L)	60.0	646.0	1994.0	>4000.0	>4000.0	>4000.0	1407.0	400.0		46
Alanine Transaminase (5–33 U/L)	23.0	243.9	887.8	2502	3210.3	2718.4	1865.1	1175.5		121.9
Ferritin (8.4–181.9 ng/mL)				8323.8	7135.9	3116.9	1236.7			186.9
C Reactive Protein (0–0.5 mg/dL)	0.2	0.14		2.65		4.62				0.71
Fibrinogen (156–400 mg/dL)		63	121	151	229	249	282			251

## Data Availability

Data available upon request to corresponding author.

## References

[1] Cron R.Q., Goyal G., Chatham W.W. (2023). Cytokine Storm Syndrome. Annu. Rev. Med..

[2] Fajgenbaum D.C., June C.H. (2020). Cytokine Storm. N. Engl. J. Med..

[3] Millar J.E., Fanning J.P., McDonald C.I., McAuley D.F., Fraser J.F. (2016). The inflammatory response to extracorporeal membrane oxygenation (ECMO): A review of the pathophysiology. Crit. Care.

[4] Henter J.-I., Horne A., Aricó M., Egeler R.M., Filipovich A.H., Imashuku S., Ladisch S., McClain K., Webb D., Winiarski J. (2007). HLH-2004: Diagnostic and therapeutic guidelines for hemophagocytic lymphohistiocytosis. Pediatr. Blood Cancer.

[5] Fardet L., Galicier L., Lambotte O., Marzac C., Aumont C., Chahwan D., Coppo P., Hejblum G. (2014). Development and Validation of the HScore, a Score for the Diagnosis of Reactive Hemophagocytic Syndrome. Arthritis Rheumatol..

[6] Al-Fares A., Pettenuzzo T., Del Sorbo L. (2019). Extracorporeal life support and systemic inflammation. Intensiv. Care Med. Exp..

[7] Datzmann T., Träger K. (2018). Extracorporeal membrane oxygenation and cytokine adsorption. J. Thorac. Dis..

[8] Warren O.J., Smith A.J., Alexiou C., Rogers P.L., Jawad N., Vincent C., Darzi A.W., Athanasiou T. (2009). The Inflammatory Response to Cardiopulmonary Bypass: Part 1—Mechanisms of Pathogenesis. J. Cardiothorac. Vasc. Anesth..

[9] Fortenberry J.D., Bhardwaj V., Niemer P., Cornish J.D., Wright J.A., Bland L. (1996). Neutrophil and cytokine activation with neonatal extracorporeal membrane oxygenation. J. Pediatr..

[10] Risnes I., Wagner K., Ueland T., Mollnes T., Aukrust P., Svennevig J. (2008). Interleukin-6 may predict survival in extracorporeal membrane oxygenation treatment. Perfusion.

[11] Hovels-Gurich H.H., Vazquez-Jimenez J.F., Silvestri A., Schumacher K., Minkenberg R., Duchateau J., Messmer B.J., von Bernuth G., Seghaye M.C. (2002). Production of pro-inflammatory cytokines and myocardial dysfunction after arterial switch operation in neonates with transposition of the great arteries. J. Thorac. Cardiovasc. Surg..

[12] Mahle W.T., Matthews E., Kanter K.R., Kogon B.E., Hamrick S.E., Strickland M.J. (2014). Inflammatory response after neonatal cardiac surgery and its relationship to clinical outcomes. Ann. Thorac. Surg..

[13] Scrascia G., Rotunno C., Guida P., Amorese L., Polieri D., Codazzi D., Paparella D. (2014). Perioperative steroids administration in pediatric cardiac surgery: A meta-analysis of randomized controlled trials. Pediatr. Crit. Care Med..

[14] Dieleman J.M., van Paassen J., van Dijk D., Arbous M.S., Kalkman C.J., Vandenbroucke J.P., van der Heijden G.J., Dekkers O.M. (2011). Prophylactic corticosteroids for cardiopulmonary bypass in adults. Cochrane Database Syst. Rev..

[15] Poli E.C., Alberio L., Bauer-Doerries A., Marcucci C., Roumy A., Kirsch M., De Stefano E., Liaudet L., Schneider A.G. (2019). Cytokine clearance with CytoSorb^®^ during cardiac surgery: A pilot randomized controlled trial. Crit. Care.

[16] Bernardi M.H., Rinoesl H., Dragosits K., Ristl R., Hoffelner F., Opfermann P., Lamm C., Preißing F., Wiedemann D., Hiesmayr M.J. (2016). Effect of hemoadsorption during cardiopulmonary bypass surgery—A blinded, randomized, controlled pilot study using a novel adsorbent. Crit. Care.

[17] Eloseily E.M., Weiser P., Crayne C.B., Haines H., Mannion M.L., Stoll M.L., Beukelman T.T., Atkinson T.P., Cron R.Q. (2020). Benefit of anakinra in treating pediatric secondary hemophagocytic lymphohistiocytosis. Arthritis Rheumatol..

[18] Durand M., Troyanov Y., Laflamme P., Gregoire G. (2010). Macrophage Activation Syndrome Treated with Anakinra. J. Rheumatol..

[19] Mehta P., Cron R.Q., Hartwell J., Manson J.J., Tattersall R.S. (2020). Silencing the cytokine storm: The use of intravenous anakinra in haemophagocytic lymphohistiocytosis or macrophage activation syndrome. Lancet Rheumatol..

[20] Bami S., Vagrecha A., Soberman D., Badawi M., Cannone D., Lipton J.M., Cron R.Q., Levy C.F. (2020). The use of anakinra in the treatment of secondary hemophagocytic lymphohistiocytosis. Pediatr. Blood Cancer.

[21] Cavalli G., De Luca G., Campochiaro C., Della-Torre E., Ripa M., Canetti D., Oltolini C., Castiglioni B., Din C.T., Boffini N. (2020). Interleukin-1 blockade with high-dose anakinra in patients with COVID-19, acute respiratory distress syndrome, and hyperinflammation: A retrospective cohort study. Lancet Rheumatol..

[22] Monteagudo L.A., Boothby A., Gertner E. (2020). Continuous Intravenous Anakinra Infusion to Calm the Cytokine Storm in Macrophage Activation Syndrome. ACR Open Rheumatol..

[23] Kavirayani A., Charlesworth J.E.G., Segal S., Kelly D., Wilson S., Qureshi A., Blanco E., Weitz J., O’Shea D., Bailey K. (2020). The Lazarus effect of very high-dose intravenous anakinra in severe non-familial CNS-HLH. Lancet Rheumatol..

[24] Kang S., Tanaka T., Narazaki M., Kishimoto T. (2019). Targeting Interleukin-6 Signaling in Clinic. Immunity.

[25] Gupta S., Wang W., Hayek S.S., Chan L., Mathews K.S., Melamed M.L., Brenner S.K., Leonberg-Yoo A., Schenck E.J., Radbel J. (2021). Association Between Early Treatment with Tocilizumab and Mortality Among Critically Ill Patients With COVID-19. JAMA Intern. Med..

[26] Lewis T.C., Arnouk S., Toy B., Geraci T.C., Carillo J.A., Chang S.H., Moazami N., Kon Z.N., Smith D.E. (2022). Tocilizumab Accelerates Recovery in Patients with Severe COVID-19 Pneumonia on Venovenous Extracorporeal Membrane Oxygenation. ASAIO J..

